# The Treatment of Pulmonary Consumption

**Published:** 1896-09

**Authors:** 


**The Treatment of Pulmonary Consumption.** By VINCENT DORMER HARRIS, M.D., and EDWIN CLIFFORD BEALE, M.B. Pp. viii., 483. London: H. K. Lewis. 1895.

The treatment of pulmonary consumption has undergone many and great changes during the last decade: formerly the diagnosis having been established, the unfortunate patient was left to his fate so far as any curative measures were concerned. *Mais nous avons changé tout cela.* Now it requires a volume such as this to give a fairly full review of the subject, and even then it might be said that some of the points are only very lightly touched upon. For instance, the use of antiseptic injections made into the lung itself has been largely adopted both on the Continent and in America; but the whole subject is here adversely criticised and dismissed in a dozen lines. As regards the use of creasote, the authors are of opinion that its effects upon tubercular disease appear to have been altogether exaggerated, and they hold that creasote cannot be considered to be either so powerful for good or for evil as has been supposed. This experience is so at variance with that of most modern observers, who hold that of all the drugs which have been employed in the treatment of tubercular affections, creasote (not, as the authors say, cod liver oil) stands first, that we have endeavoured to ascertain on what grounds the authors formed their conclusion. It is generally admitted that the value of the drug varies in proportion to the dose which the patient is able to take; but here we find that "it is seldom necessary, or indeed advisable, to give more than twenty to thirty minims, and, generally speaking, six to fifteen minims in the twenty-four hours is *a sufficient dose*" (the italics are ours). We wonder in what sense it is sufficient. Clearly in the authors' experience the results were not what they would wish, and yet the dose was considered to be sufficient, *i.e.* sufficient to satisfy the views of the authors, but not sufficient for the needs of the patient. We think that a more liberal use of the drugs of the creasote type might give more satisfactory results, and prove that these drugs are both potent for good, even more good than the authors lead us to expect, and for harm when given in very large doses in the late stages of the disease. We have rarely any difficulty in getting patients to take sixty or ninety minims of the pure drug in twenty-four hours, and the more completely we can saturate the patient the more likely shall we be to modify the development and growth of the bacilli in the lungs. Doses such as this are, however, not to be given when the degree of prostration is great, as they produce toxic results and add to the prostration: in cases of this kind we have sometimes thought that they accelerated the death of the patient.

We must refrain from further criticism of a book which is a mine of wealth, and which is probably the most complete on the subject which has ever been written in this or any other language. It is a book to be first studied, then kept at hand for reference, and concerning which the opinion of any reviewer should count for little or nothing.

